# Attention-enhanced hybrid deep learning model for robust mango leaf disease classification via ConvNeXt and vision transformer fusion

**DOI:** 10.3389/fpls.2025.1638520

**Published:** 2025-08-01

**Authors:** Ebru Ergün

**Affiliations:** Department of Electrical and Electronics Engineering, Faculty of Engineering and Architecture, Recep Tayyip Erdogan University, Rize, Türkiye

**Keywords:** agricultural imaging, ConvNeXt, cross-modal dynamic fusion, disease classification, mango leaf, vision transformer

## Abstract

Mango is a crop of vital agronomic and commercial importance, particularly in tropical and subtropical regions. Accurate and timely identification of foliar diseases is essential for maintaining plant health and ensuring sustainable agricultural productivity. This study proposes MangoLeafCMDF-FAMNet (cross-modal dynamic fusion with feature attention module (FAM) network), an advanced, hybrid, deep-learning framework designed for the multi-class classification of mango leaf diseases. The model combines two state-of-the-art feature extractors, ConvNeXt and Vision Transformer, to capture local fine-grained textures and global contextual semantics simultaneously. To further improve feature discrimination, a FAM inspired by squeeze-and-excitation networks is integrated into each stage of the backbone. This module adaptively recalibrates channel-wise feature responses to highlight disease-relevant cues while suppressing irrelevant background noise. A novel cross-modal dynamic fusion strategy unifies the complementary strengths of both branches, resulting in highly robust and discriminative feature embeddings. The proposed model was rigorously evaluated using comprehensive metrics such as classification accuracy (CA), recall, precision, Matthews correlation coefficient (MCC) and Cohen’s kappa score on three benchmark datasets: MangoLeafDataset1 (8 classes), MangoLeafDataset2 (5 classes) and MangoLeafDataset3 (8 classes). The experimental results consistently demonstrate the superiority of MangoLeafCMDF-FAMNet over the existing baseline models. It achieves exceptional CA values of 0.9978, 0.9988 and 0.9943 across the respective datasets, alongside strong MCC and Cohen’s kappa scores. These results highlight the effectiveness and generalizability of the proposed framework for automated mango leaf disease diagnosis and contribute to advancing deep learning applications in precision plant pathology.

## 1 Introduction

The mango is of great agronomic and economic importance, particularly in tropical and subtropical regions, where it is one of the most widely cultivated fruit crops (Zhang et al., 2025). However, mango plants’ productivity and health are persistently threatened by foliar diseases, which impair photosynthetic efficiency and lead to significant reductions in yield and fruit quality. Although traditional approaches to disease diagnosis are still widely used, they often involve subjective assessments, delayed response times and a heavy dependence on expert knowledge. These limitations highlight the urgent need for automated, accurate and scalable diagnostic tools to support the timely and objective management of mango diseases.

In recent years, deep learning (DL) techniques have transformed plant disease detection by enabling complex, hierarchical patterns to be extracted directly from image data. Unlike conventional handcrafted approaches, DL models have demonstrated superior performance in various agricultural vision tasks. However, classifying mango leaf diseases is challenging due to their intricate visual symptoms, similarities between classes and variations within classes that frequently occur across disease types (Shehu et al., 2025; Wei et al., 2025). These complexities necessitate the development of more advanced architectures that can effectively learn both fine-grained local textures and high-level semantic features.

In response to these challenges, we propose MangoLeafCMDF-FAMNet (cross-modal dynamic fusion (CMDF) with feature attention module (FAM) network), a novel hybrid DL framework specifically designed for multi-class mango leaf disease classification. This architecture integrates ConvNeXt and Vision Transformer (ViT) as dual feature extractors, combining the strengths of convolutional inductive biases and transformer-based global attention mechanisms. The proposed model uses a CMDF strategy to combine texture- and semantic-level information into a coherent, enriched feature representation space. To further enhance feature expressiveness, a FAM, inspired by squeeze-and-excitation (SE) networks, is incorporated at each stage. This module adaptively recalibrates channel-wise feature responses to prioritize disease-relevant patterns while suppressing irrelevant background noise.

To comprehensively evaluate the performance of MangoLeafCMDF-FAMNet, we conduct extensive experiments on three publicly available mango leaf disease datasets—MangoLeafDataset1 (8 classes), MangoLeafDataset2 (5 classes), and MangoLeafDataset3 (8 classes). The model’s effectiveness is quantified using multiple evaluation metrics, including classification accuracy (CA), recall (RCL), precision (PRC), Matthews correlation coefficient (MCC), and Cohen’s kappa score. The experimental results consistently demonstrate that the proposed method significantly outperforms conventional baseline models across all datasets, achieving high CA and strong correlation measures.

The main contributions of this work are as follows:

- We introduce MangoLeafCMDF-FAMNet, a novel hybrid DL architecture that combines ConvNeXt and ViT with FAM for enhanced hierarchical feature representation.
- We design a CMDF strategy that effectively fuses local texture information with global contextual features, leading to more robust representations.
- We perform a thorough evaluation across multiple public datasets, establishing the superior classification performance of the proposed model in terms of CA, RCL, PRC, MCC, and kappa.
- We offer a generalizable and scalable framework with practical implications for automated mango disease diagnosis, and the potential for adaptation to other plant disease classification tasks.

The remainder of this paper is structured as follows: Section 2 provides a thorough review of existing research for plant disease detection. Section 3 outlines the materials and methods employed in this study, providing detailed descriptions of the dataset and the proposed hybrid deep learning and feature selection framework. Section 4 reports the experimental results, alongside performance evaluation metrics and comparative analyses. Section 5 concludes the paper by summarizing the main findings and highlighting potential future research directions. Finally, Section 6 critically discusses the study’s limitations and underlying assumptions, as well as its practical implications for real-world agricultural applications.

## 2 Review of existing approaches

Recent advances in computer vision and DL have significantly accelerated the development of automated tools for plant disease diagnosis. convolutional neural networks (CNNs) and transformer-based models, especially ViTs, have emerged as dominant paradigms in plant pathology research due to their ability to extract discriminative spatial and semantic patterns from complex visual data (Chen et al., 2024). However, existing studies on mango leaf disease classification have faced multiple challenges that limit their practical utility and generalizability. Among these studies, Rao et al. (2021) initiated one such effort by leveraging the PlantVillage dataset, applying the AlexNet architecture, and achieving classification accuracies of 0.9900 for grape leaves and 0.8900 for mango leaves. Building on this, Arivazhagan and Ligi (2018) utilized a CNN-based approach on a six-class mango dataset and attained a commendable accuracy of 0.9667. In contrast, Mia et al. (2020) combined artificial neural networks and support vector machines, achieving 0.8000 accuracy in detecting four disease classes and healthy leaves. A more advanced ensemble strategy was introduced by Gautam et al. (2024), who developed a Stacked Ensemble Deep Neural Network that integrated multiple DNNs with classical ML classifiers, yielding a high accuracy of 0.9857 across eight disease classes. Similarly, Saleem et al. (2021a) investigated disease detection using canonical correlation analysis (CCA)-based feature fusion and found cubic SVM to deliver the highest performance. In a follow-up study, Saleem et al. (2021b) further proposed the FrCNet model for lesion segmentation and, after combining it with CCA feature fusion and classification via quadratic and cubic SVMs, achieved 0.9890 accuracy for binary disease-versus-healthy discrimination. Continuing the exploration of CNN variants, Varma et al. (2025) benchmarked several pretrained architectures, with InceptionV3 achieving the highest accuracy at 0.9987. Meanwhile, Patel et al. (2024) introduced a hybrid framework combining Total Variation Filter-based variational mode decomposition with DenseNet121 and VGG-19, achieving 0.9885 CA. This fusion approach notably improved feature interpretability and robustness against noise. Hossain et al. (2024) evaluated ViTs against well-established CNNs and proposed an optimized DeiT-based model, which outperformed all compared methods with a CA of 0.9975. Similarly, Mahmud et al. (2024) proposed DenseNet78, a lightweight variant of DenseNet tailored for mango leaf disease classification, reporting accuracies of 0.9947 for healthy and 0.9944 for diseased leaves. In practical implementations, Puranik et al. (2024) retrained MobileNetV3 on the MangoLeafBD dataset and embedded it within a mobile application, reaching 0.9800 accuracy and enabling real-time field diagnosis. Singh et al. (2024) adopted a transfer learning approach and proposed the DTLD model, which demonstrated strong multi-class classification performance with a peak accuracy of 0.9976 on a 4000-image dataset. Expanding on comparative model analysis, Bairwa et al. (2024) assessed multiple deep networks, finding ResNet50 to deliver the highest accuracy at 0.9912. Complementarily, Pratap and Kumar (2024) designed a CNN-based system incorporating transfer learning from VGG-16, GoogLeNet, MobileNet, YOLOv8, and EfficientNet, enabling effective classification of several mango diseases including Anthracnose, Gall Midge, and Powdery Mildew. Finally, Pahati et al. (2025) trained a Google Teachable Machine model on 4000 annotated images, obtaining an accuracy of 0.9960 and demonstrating high potential for democratized, user-friendly disease recognition platforms.

Most conventional CNN-based models, although capable of capturing local textures, fall short in modeling long-range dependencies—a critical requirement for accurately distinguishing visually similar diseases with subtle morphological variations. Transformer-based methods, while excellent at global context modeling, often lack the inductive biases necessary for fine-grained feature localization. As such, stand-alone CNN or ViT models struggle to deliver optimal performance across varying environmental conditions and disease stages observed in agricultural settings. For example, the study by Alamri et al. (2025) proposed a dual-branch architecture combining ConvNeXt and ViT to detect mango leaf and fruit diseases separately using the MangoLeafBD and SenMangoFruitDDS datasets. Their model achieved promising accuracy levels of 99.87% and 98.40% respectively, demonstrating the value of hybrid architectures in plant disease classification. However, their method did not incorporate any explicit attention mechanism to recalibrate the feature importance across network layers. Furthermore, their architecture processed the outputs of ConvNeXt and ViT using a static fusion approach, which may limit the adaptability of feature interactions during training.

By contrast, our proposed MangoLeafCMDF-FAMNet framework introduces several significant improvements to the original design. Firstly, inspired by SE networks, we incorporated a FAM at each stage to dynamically recalibrate channel-wise features. This enables the model to selectively emphasize disease-relevant information and suppress background noise. Secondly, instead of using a static feature aggregation strategy, our model uses a CMDF mechanism to adaptively combine spatial and semantic cues extracted from ConvNeXt and ViT backbones. This significantly improves the model’s representational richness and robustness.

Moreover, MangoLeafCMDF-FAMNet was rigorously evaluated on three distinct datasets encompassing both 5-class and 8-class classification tasks. Experimental results demonstrated that our model consistently outperforms traditional CNNs, ViTs, and hybrid baselines—including the model by Alamri et al. (2025)—not only in terms of CA but also across comprehensive evaluation metrics such as MCC and kappa. The superior performance of our model, particularly under multi-class, real-world conditions, underscores its potential as a scalable and generalizable solution for precision agriculture.

Importantly, foliar disease diagnosis remains a crucial but underexplored area in the literature, especially concerning tropical crops such as mango. Leaf diseases are often early indicators of plant stress and can significantly affect fruit development and overall yield. Therefore, developing robust, accurate, and field-deployable diagnostic systems for leaf disease identification is critical for achieving sustainable agricultural outcomes. Our contribution lies not only in achieving state-of-the-art performance but also in offering a practical architecture that balances accuracy, computational efficiency, and adaptability, setting a new benchmark in artificial intelligence (AI)-assisted mango disease diagnosis.

## 3 Materials and methods

### 3.1 Description of dataset

#### 3.1.1 MangoLeafDataset1

The Mango MLD dataset, (MLD1) curated by Shakib et al., served as one of the primary data sources in this study (Shakib et al., 2024). This publicly available dataset was meticulously compiled through an extensive field data acquisition campaign conducted across diverse mango orchards situated in Kushtia and Dhaka, Bangladesh. The primary objective of this collection effort was to capture high-quality, representative images of both healthy and diseased mango leaves under realistic agricultural conditions, thereby ensuring the ecological validity and practical relevance of the dataset for real-world disease classification tasks.

A total of 6,400 images were included in the dataset, uniformly distributed across eight diagnostic categories. Seven of these classes correspond to prevalent mango leaf diseases—Anthracnose, Bacterial Canker, Cutting Weevil, Die Back, Gall Midge, Powdery Mildew, and Sooty Mould—while the eighth class represents healthy leaves. To mitigate class imbalance and enable unbiased model training, each category contains exactly 800 images, making this dataset structurally balanced. The images were originally captured using an iPhone SE device at a native resolution of 3024 × 4032 pixels and subsequently downscaled to 240 × 240 pixels in JPEG format. This resizing operation was performed to reduce memory overhead without significantly compromising visual quality or diagnostic features. Crucially, no synthetic augmentation was applied to the original images, preserving the integrity and authenticity of real-world leaf textures, color gradients, and lesion morphologies.

**Figure 1:**
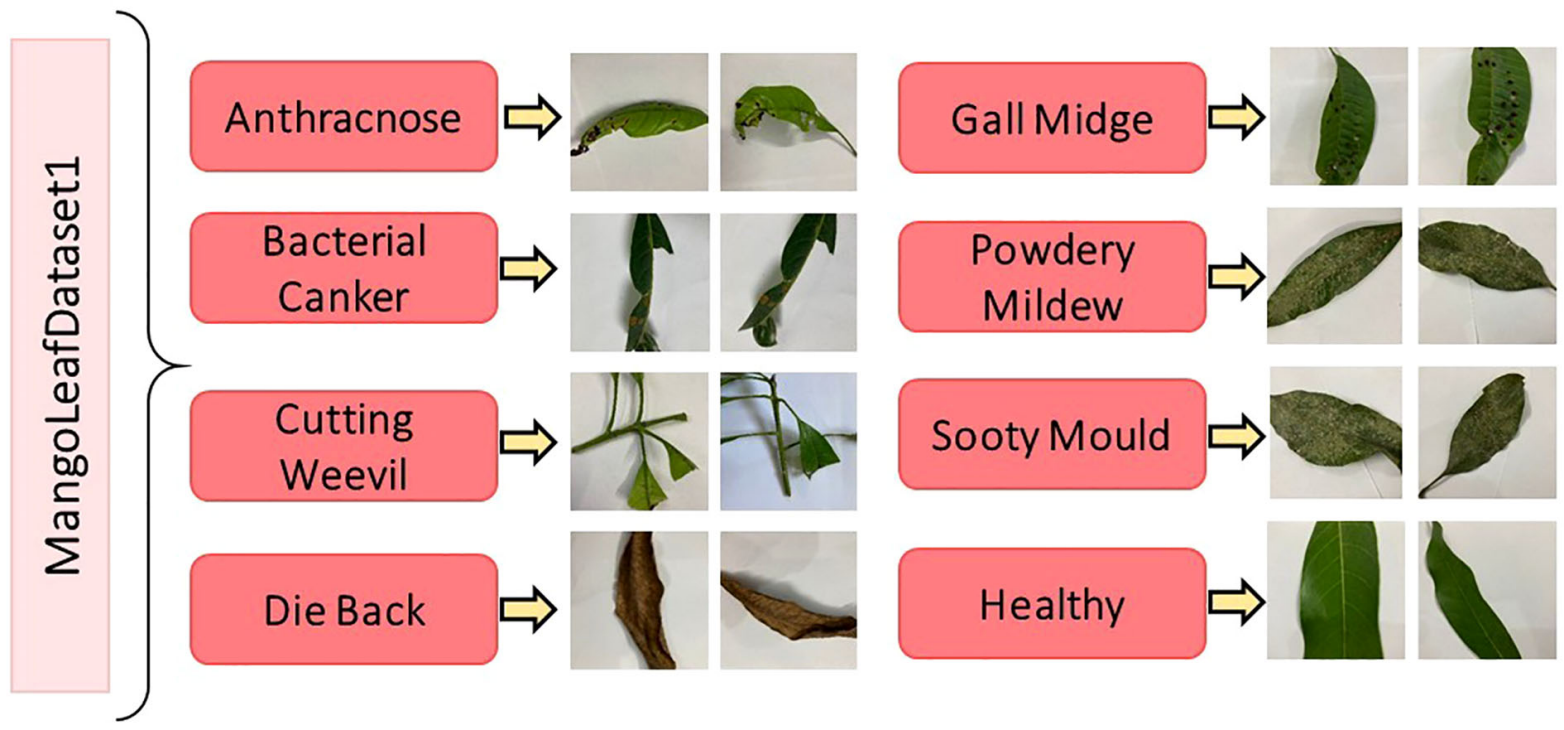
Representative images from the MLD1 dataset, illustrating both healthy mango leaves and leaves affected by seven distinct diseases (Shakib et al., 2024).

**Figure 1** presents representative samples from each disease class, providing visual insight into the morphological and pathological variations captured in the dataset. Meanwhile, the corresponding distribution of class frequencies is detailed in **Table 1**, where the uniformity of sample counts across categories is explicitly demonstrated.

**Table 1:** Class-wise distribution of images in the MLD1 (Shakib et al., 2024).

Class Name	MLD1
	Number of images
Anthracnose	800
Bacterial Canker	800
Cutting Weevil	800
Die Back	800
Gall Midge	800
Powdery Mildew	800
Sooty Mould	800
Healthy	800
Total	6400

#### 3.1.2 MangoLeafDataset2

As a complementary data source, the MangoLeafDataset2 (MLD2)—compiled and published by Nirob et al.—was incorporated into this study to further strengthen the reliability and generalizability of the proposed classification model (Nirob et al., 2024). This dataset offers a rich collection of high-resolution mango leaf images, originally captured between August 15 and August 29, 2023, in the mango cultivation fields of Supu Ashulia, Bangladesh. The data acquisition process was carried out under natural lighting and environmental conditions, ensuring that the captured leaf samples reflect real-world visual characteristics, including noise, background clutter, and variability in disease presentation.

**Figure 2:**
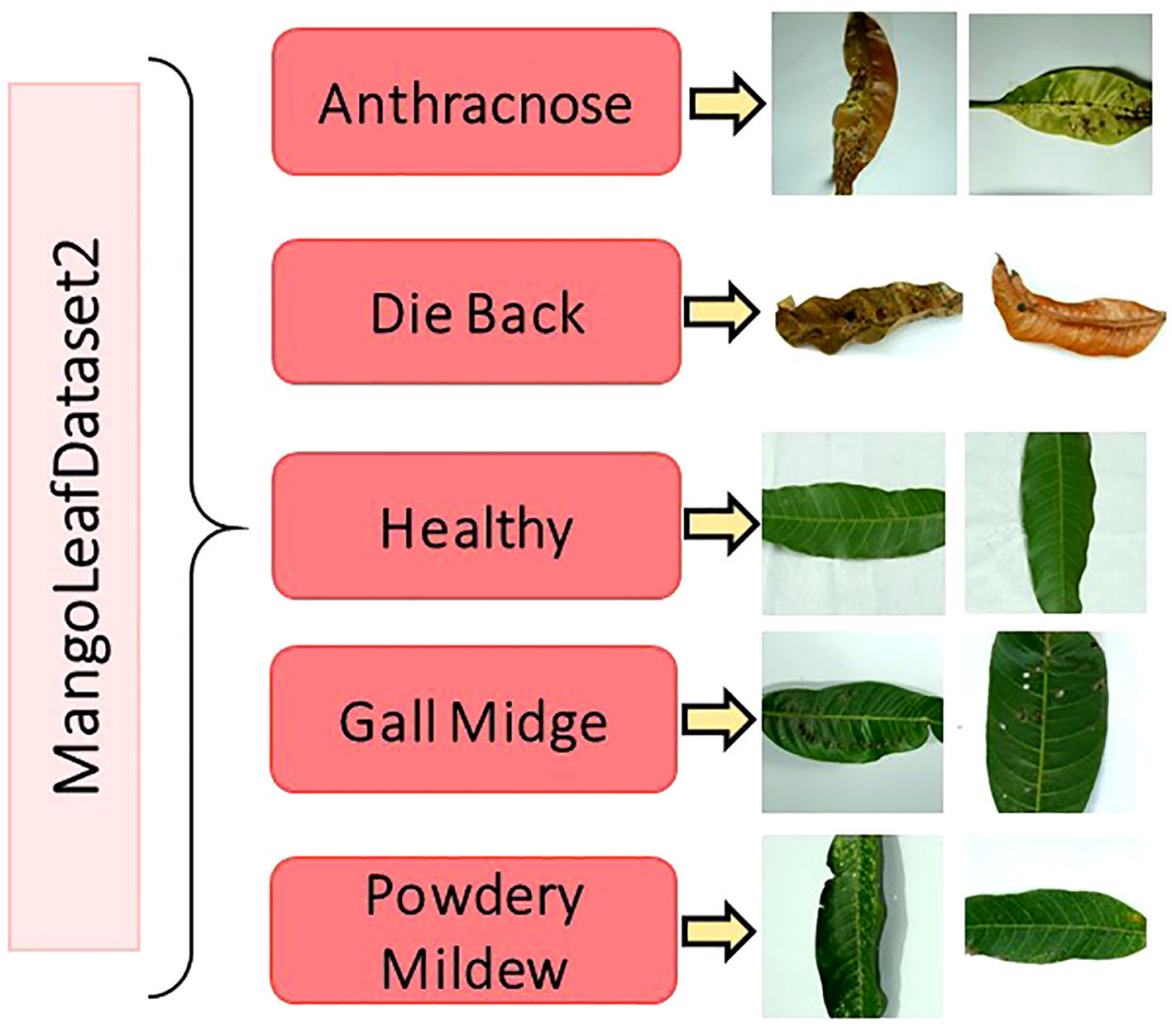
Selected image samples from the MLD2 dataset, reflecting variations in leaf color, texture, and shape across four disease classes and healthy leaves (Nirob et al., 2024).

The original dataset comprises 1,319 unique images, each with a standardized resolution of 1000 × 1000 pixels and stored in JPEG format. The dataset encompasses five key categories representing distinct pathological states of mango leaves: Anthracnose, Die Black, Gall Midge, Powdery Mildew, and Healthy. These categories were carefully selected based on the prevalence and diagnostic importance of the corresponding diseases in commercial mango production. To overcome the inherent class imbalance, present in the original dataset and to enhance the learning capability of deep models, a comprehensive data augmentation strategy was applied. Techniques such as horizontal and vertical flipping, arbitrary rotations, scaling, and mild intensity transformations were utilized to synthetically expand the dataset. As a result, each category was normalized to contain exactly 1,000 samples, thereby yielding a final augmented dataset comprising 5,000 images. **Figure 2** illustrates representative image samples from each of the five classes, providing a visual overview of the phenotypic diversity embedded within the dataset. **Table 2** summarizes the distribution of both original and augmented images per class.

**Table 2:** Augmented image counts for each class in the MLD2 (Nirob et al., 2024).

Class Name	MLD2
	Number of images
Anthracnose	1000
Die Back	1000
Gall Midge	1000
Powdery Mildew	1000
Healthy	1000
Total	5000

#### 3.1.3 MangoLeafDataset3

To further enhance the robustness and cross-dataset generalizability of the proposed classification framework, this study integrated the MangoLeafDataset3 (MLD3), meticulously curated by Rahman et al. and publicly released in November 2024 (Rahman et al., 2024). This dataset serves as a significant and diverse benchmark resource for intelligent agricultural analysis, particularly in the field of mango leaf disease recognition. The data acquisition phase was carried out over a period of 20 consecutive days, from October 15 to November 4, 2024, in two distinct agroecological regions of Bangladesh—Kashinathpur (Pabna) and Changao (Savar, Dhaka)—to capture a wide spectrum of environmental and disease conditions. The dataset is composed of two main subsets: 2,336 raw images captured under natural lighting conditions using mobile phone cameras, and 12,730 synthetically augmented images generated through comprehensive data enhancement techniques. These augmentations include but are not limited to affine transformations, horizontal/vertical flips, minor brightness and contrast shifts, and random cropping, all designed to introduce variability and enrich the dataset’s learning potential without compromising biological authenticity. All images are categorized into eight distinct classes, representing seven pathological categories—Anthracnose, Bacterial Canker, Cutting Weevil, Die Back, Gall Midge, Powdery Mildew, and Sooty Mould—along with one Healthy class.

**Figure 3:**
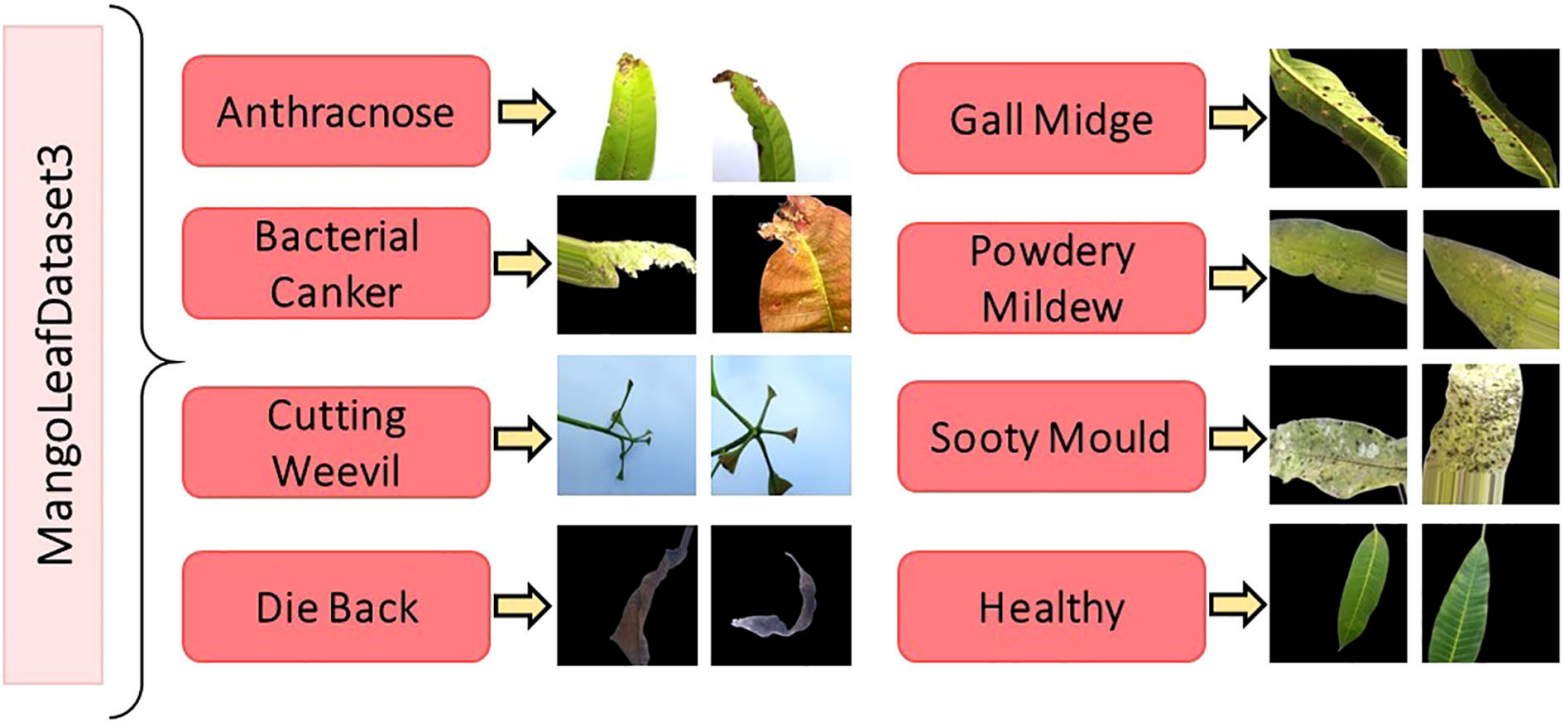
Image samples from the MLD3 dataset highlighting intra-class variability and visual diversity, which pose additional challenges for robust disease classification (Rahman et al., 2024).

The original class distribution, prior to augmentation, is intentionally preserved to reflect natural disease occurrence rates. However, the expanded dataset introduces balance and diversity necessary for training deep neural models effectively. A comprehensive breakdown of the image count per class is provided in **Table 3**, while **Figure 3** visually showcases representative samples from each class, highlighting inter-class visual variability and intra-class complexity.

**Table 3:** Augmented image counts per class in the MangoLeafDataset3 (Rahman et al., 2024).

Class Name	MLD3
	Number of images
Anthracnose	1749
Bacterial Canker	2534
Cutting Weevil	1583
Die Back	1280
Gall Midge	2233
Powdery Mildew	776
Sooty Mould	1325
Healthy	1250
Total	12730

### 3.2 Research methodology framework

**Figure 4:**
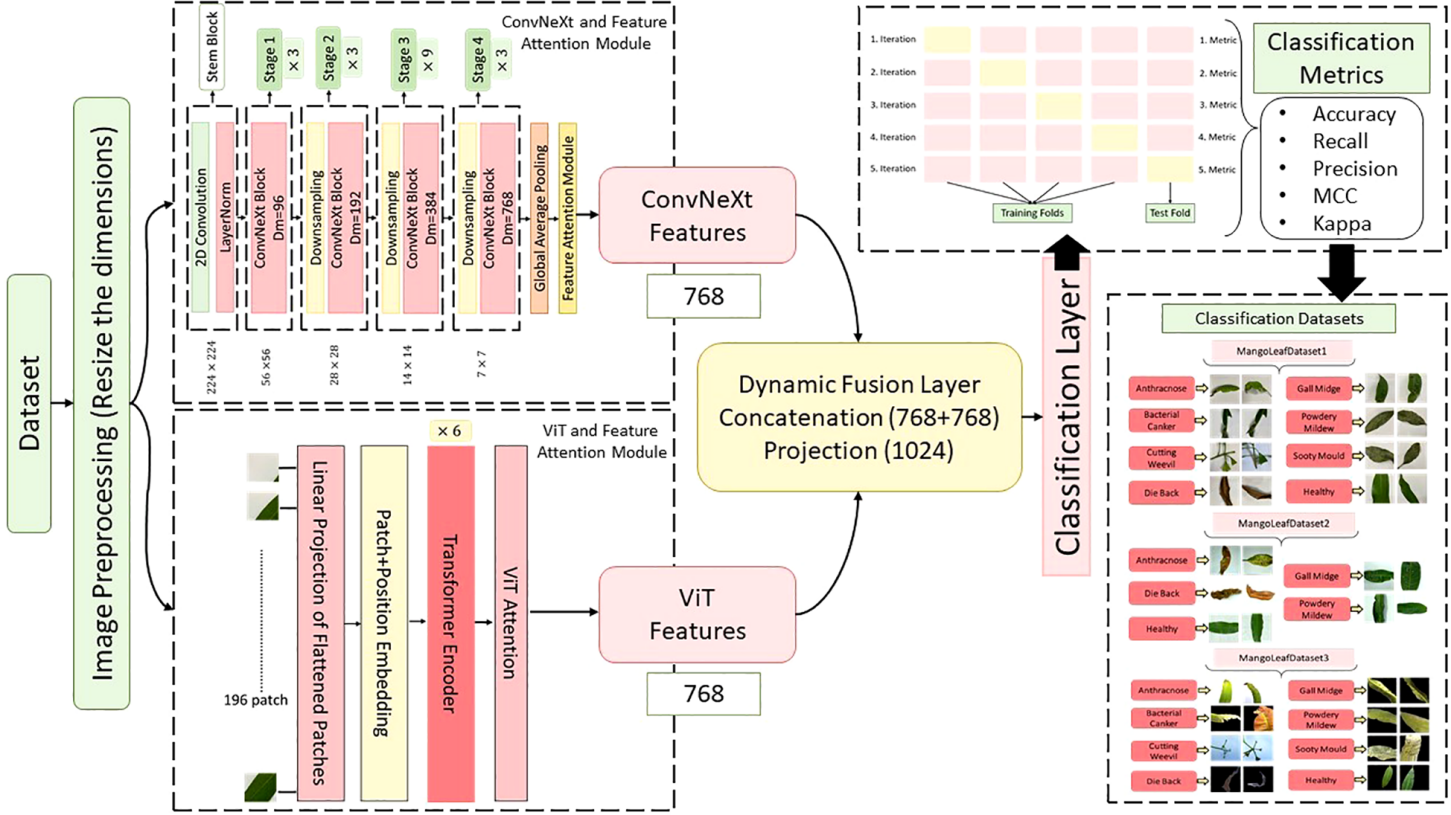
Overview of the proposed MangoLeafCMDF-FAMNet architecture, illustrating the dual-branch design consisting of ConvNeXt and ViT, integrated with FAM and a CMDF mechanism for feature integration and disease classification.

In this study, a novel hybrid DL architecture named MangoLeafCMDF-FAMNet was proposed to address the complex problem of mango leaf disease classification. The methodology capitalized on the complementary strengths of two advanced feature extractors—ConvNeXt and ViT—to capture fine-grained texture details as well as global contextual dependencies inherent in leaf imagery. The overall flow of the proposed method is illustrated in **Figure 4**. To train and validate the proposed framework, three publicly available datasets were employed: the 8-class MLD1, 5-class MLD2, and 8-class MLD3. All image samples were preprocessed with standard normalization and resized to a uniform resolution of 224 × 224 pixels to ensure consistency across training folds. Data augmentation techniques were deliberately excluded to assess the raw generalization power of the model.

The MangoLeafCMDF-FAMNet model integrated a ConvNeXt-Tiny backbone pre-trained on ImageNet as its local feature extractor. Its final classification head was removed and replaced with a global average pooling layer, followed by a custom FAM inspired by SE networks. In parallel, a lightweight ViT was employed to model long-range semantic interactions, with its outputs refined through a 1D feature-wise attention mechanism designed to amplify class-relevant representations. After extracting the deep features from both ConvNeXt and ViT branches, a CMDF strategy was applied. This strategy concatenated the learned embeddings and passed them through a projection layer, resulting in a unified 1024-dimensional representation. The fused vector was then forwarded to a fully connected classifier to generate final class predictions.

The performance evaluation of the proposed model was conducted using a stratified 5-fold cross-validation protocol (5-FCVP) to ensure reliable and unbiased assessment. In each fold, the dataset was partitioned into distinct training and validation subsets while preserving class distribution. During training, the model parameters were optimized using the AdamW optimizer, configured with a learning rate of 0.00005 and a weight decay coefficient of 0.0001 to promote generalization. The cross-entropy loss function served as the optimization objective, guiding the network’s learning process. For each validation phase, a comprehensive set of evaluation metrics was computed, including CA, RCL, PRC, MCC, and kappa, to provide a multi-faceted performance analysis. Additionally, confusion matrices were generated for each fold to reveal class-specific prediction behaviors. To qualitatively investigate the separability of learned features, high-dimensional embeddings were projected into a two-dimensional space using t-distributed stochastic neighbor embedding (t-SNE), offering visual insight into the model’s discriminative capability.

### 3.3 ConvNeXt backbone architecture

In this study, ConvNeXt was selected as one of the core backbone networks of the proposed CMDF-Net due to its ability to effectively extract hierarchical features from input images by leveraging the design principles of both ResNet and transformer-based architectures. ConvNeXt is a convolutional neural network that modernizes the classic ResNet architecture through architectural refinements inspired by the success of ViTs, achieving a competitive balance between performance and efficiency in visual recognition tasks.

ConvNeXt comprises multiple stages, each containing a sequence of blocks designed to progressively capture low-level to high-level semantic features (Fu et al., 2025). Each block within ConvNeXt replaces the traditional bottleneck structure of ResNet with a streamlined stack of operations, composed of a depthwise convolution (DWConv), a layer normalization (LN), a pointwise convolution (1×1 Conv), and a GELU activation function. Mathematically, the core block of ConvNeXt can be formulated as follows. Let $x\in R^{H\times W\times C}$ represent the input tensor, where *H*, *W*, and *C* denote the height, width, and number of channels, respectively. The transformation f(x) within a ConvNeXt block is defined as Equation 1 (Ford et al., 2025).


(1) $$\text{f}(\text{x})={\text{W}}_{2}GELU(LN({\text{W}}_{1}\cdot DWConv(x)))$$

where, DWConv represents the depthwise convolutional operation with a kernel size of 7×7, designed to capture spatial correlations within each channel independently. $W_{1}$ ​ and $W_{2}$ ​ denote pointwise (1×1) convolution weights that project the input and output feature spaces. LN is the layer normalization function, which stabilizes training and accelerates convergence. GELU stands for Gaussian error linear unit, providing smoother activation compared to ReLU.

In this implementation, ConvNeXt was configured with the “ConvNeXt-Tiny” variant to ensure a balanced trade-off between computational cost and feature extraction capability. The network was divided into four stages, where each stage contains multiple ConvNeXt blocks and concludes with a downsampling layer that reduces the spatial resolution while increasing the channel dimension (Lu et al., 2025). The channel dimensions across the stages were configured as [96,192,384,768] , and the number of blocks per stage were [3,3,9,3] , respectively. To further enhance the representational capacity of ConvNeXt, we integrated a FAM at the output of each stage. Inspired by the SE networks, this module adaptively recalibrates the feature maps along the channel dimension. The mechanism operates in three steps: squeeze, excitation, and reweighting. Let $U\in R^{H\times W\times C}$ in denote the output feature map of a ConvNeXt stage. The channel-wise global descriptor z $\in R^{C}$ is obtained via global average pooling given as Equation 2 (Tao et al., 2022).


(2) $${\text{z}}_{\text{c}}=\frac{1}{\text{H}\times \text{W}}\sum_{\text{i}=1}^{\text{H}}\sum_{\text{j}=1}^{\text{W}}{\text{U}}_{\text{i},\;\text{j},\;\text{c}}$$

Then, the excitation step applies a gating mechanism using two fully connected layers with non-linearity is shown in Equation 3.


(3) $$\text{s}=\text{σ}({\text{W}}_{2}\cdot \text{δ}({\text{W}}_{1}\cdot \text{z}))$$

where, $W_{1}\in R^{\frac{C}{r}\times C}$ and $W_{2}\in R^{C\times \frac{C}{r}}$ are the weights of the fully connected layers, δ is the ReLU activation function, σ is the sigmoid activation function, r is the reduction ratio (set to 16 in this study) controlling the bottleneck. Finally, the recalibrated feature map $\hat{U}$ is obtained by channel-wise multiplication is given in Equation 4.


(4) $${\hat{\text{U}}}_{\text{c}}={\text{s}}_{\text{c}}\cdot {\text{U}}_{\text{c}}$$

**Figure 5:**
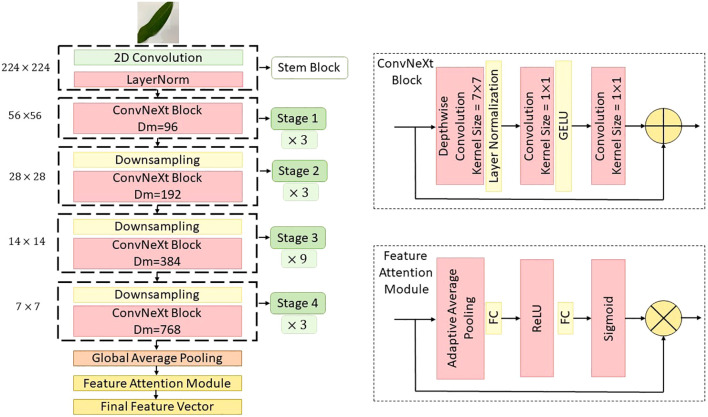
Detailed schematic of the ConvNeXt backbone as implemented within the MangoLeafCMDF-FAMNet framework, showing key stages of convolutional feature extraction and attention recalibration.

This attention mechanism allows the network to selectively emphasize informative features while suppressing less useful ones, thereby boosting the model’s ability to focus on disease-related patterns in mango leaf images. The output feature maps from all ConvNeXt stages, enhanced by their respective attention modules, are then passed to the fusion layer as shown **Figure 5**.

### 3.4 Vision transformer backbone architecture

As a complementary backbone to ConvNeXt, the ViT was employed in CMDF-Net to exploit the global context modeling capabilities of self-attention mechanisms. ViT treats images as sequences of non-overlapping patches, analogous to tokens in natural language processing, and applies standard Transformer encoders to capture long-range dependencies and global feature representations, which are crucial for identifying disease patterns distributed across different regions of mango leaves (Ergün, 2025). The input image $x\in R^{H\times W\times C}$ is first divided into a grid of N patches of size P×P where $=\frac{HW}{P^{2}}$ . Each patch is flattened and projected into a D-dimensional embedding space through a linear layer as given Equation 5.


(5) $${\text{z}}_{0}^{\text{i}}=\text{E}\cdot \text{flatten}(x^{i})+p_{i},\;\;\;\;\;\;\;\;\;\;\;\text{i}=1,\;2,\ldots .,\text{N}$$

where, $x^{i}$ is the $i^{th}$ image patch, $\;E\in R^{D\times (P^{2}\cdot C)}$ is the learnable patch embedding matrix, $P_{i}\in R^{D}$ is the learnable positional embedding added to each patch token to retain spatial information.

In addition, a learnable classification token $z_{0}^{\left[cls\right]}\in R^{D}$ is prepended to the patch sequence, which serves as the aggregated representation of the input image after processing through the Transformer layers (Kamal et al., 2025). The final input to the Transformer encoder is shown Equation 6.


(6) $${\text{Z}}_{0}=[{\text{z}}_{0}^{\left[cls\right]},\;{\text{z}}_{0}^{1},\;{\text{z}}_{0}^{2},\ldots \ldots .,\;{\text{z}}_{0}^{\text{N}]}]\in {\text{R}}^{(\text{N}+1)\times \text{D}}$$

The Transformer encoder consists of L identical layers, each composed of a multi-head self-attention (MSA) mechanism followed by a position-wise feed-forward network (FFN). Each layer also includes residual connections and layer normalization as seen Equations 7 and 8 (Lu et al., 2025).


(7) $$\hat{{\text{Z}}_{\text{l}}}=\text{MSA}(\text{LN}({\text{Z}}_{\text{l}-1}))+{\text{Z}}_{\text{l}-1}$$

(8) $${\text{Z}}_{\text{l}}=\text{FNN}(\text{LN}(\hat{{\text{Z}}_{\text{l}}}))+\hat{{\text{Z}}_{\text{l}}},\;\;\;\;\;\;\;\text{l}=1,\ldots .,\text{L}$$

Here, the MSA operation splits the input into h heads and performs scaled dot-product attention in parallel as given in Equation 9.


(9) $$\text{Attention}(\text{Q},\;\text{K},\;\text{V})=\;\text{softmax}(\frac{\text{Q}K^{T}}{\sqrt{d_{k}}})\text{V}$$

where $Q,\;K,\;V\in R^{(N+1)\times d_{k}}$ are the query, key, and value matrices computed from the input via learned linear projections, and dk=Dh is the dimensionality of each head. The softmax function transforms the similarity scores into a probability distribution as given the Equation 10.


(10) $$\text{softmax}({\text{a}}_{\text{i}})=\frac{{\text{e}}^{{\text{a}}_{\text{i}}}}{\sum_{\text{j}=1}^{\text{n}}{\text{e}}^{{\text{a}}_{\text{i}}}}\;\;\;\;\;\;\;\text{i}=1,2,\ldots ,\text{n}$$

The ViT backbone used in this study was based on the “ViT-Base” variant, configured with the following parameters: patch size P =16, embedding dimension D =768, number of transformer layers L =12, number of attention heads h =12, feed-forward dimension $d_{ff}$ =3072.

To further enrich the discriminative capability of ViT features, we introduced a FAM at the output of the transformer. This module, similar to the one used in ConvNeXt, emphasizes important channels in the output embedding of the classification token $z_{L}^{\left[cls\right]}$ , based on global channel context. Given the final Transformer output $Z_{L}$ , the class token vector $z_{L}^{\left[cls\right]}\in R^{D}$ is passed through a SE-inspired gating mechanism as shown in Equations 11 and 12 (Padshetty and Umashetty, 2024).


(11) $$\text{s}=\text{σ}({\text{W}}_{2}\cdot \text{δ}({\text{W}}_{1}\cdot z_{L}^{\left[cls\right]}))$$

(12) $${\hat{\text{z}}}_{\text{L}}^{\left[cls\right]}=\text{s}\cdot z_{L}^{\left[cls\right]}$$

**Figure 6:**
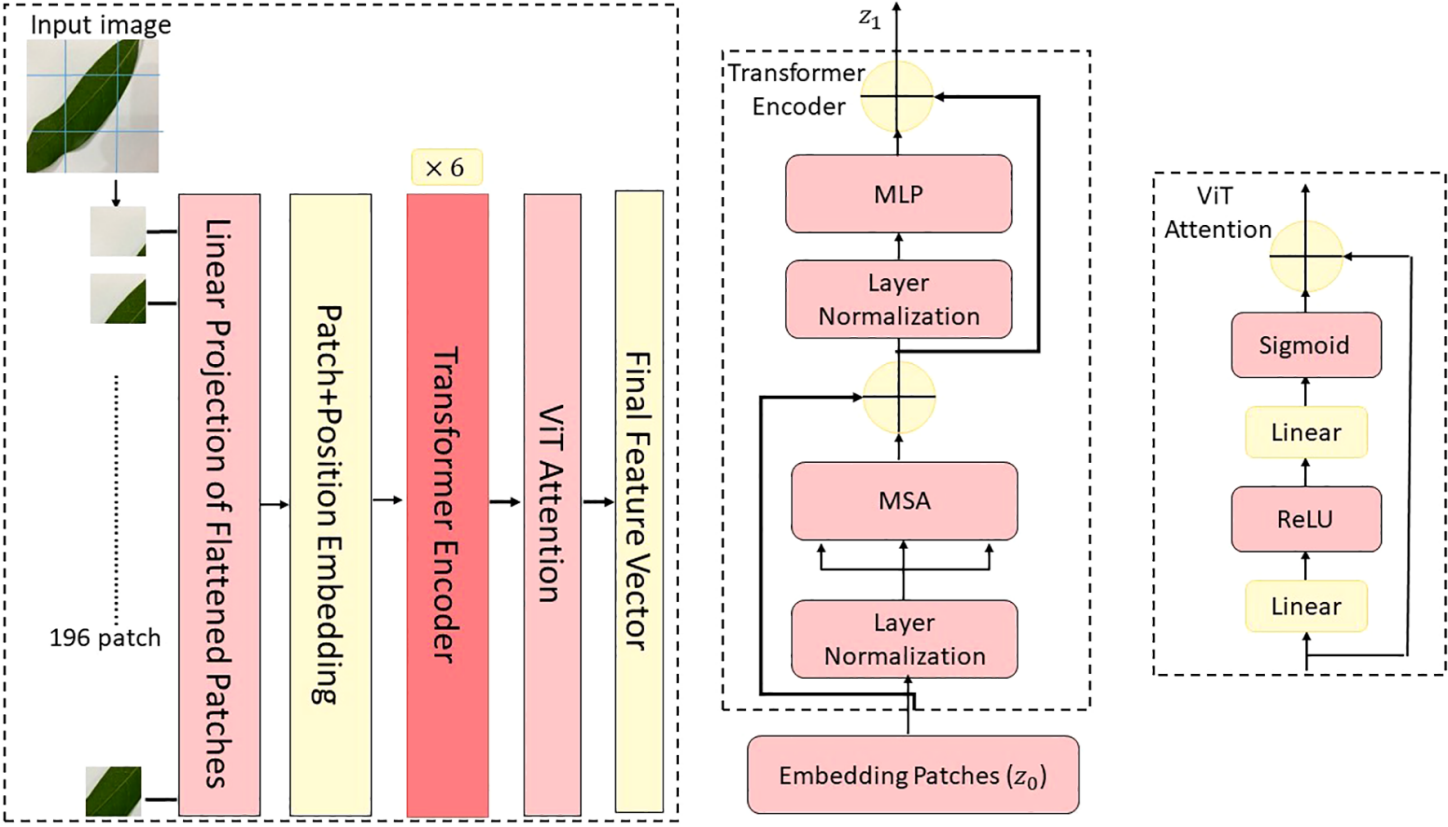
Architectural illustration of the ViT module employed in the proposed model, depicting patch embedding, transformer encoding, and output token generation stages.

This attention-weighted representation $z_{L}^{\left[cls\right]}$ captures the globally aggregated and recalibrated semantic information, which is later fused with the multiscale ConvNeXt features during the dynamic fusion stage of CMDF-Net as seen **Figure 4**. Also, the global modeling capacity of ViT given as **Figure 6** robust feature extraction across both local textures and global structures in diseased mango leaf images.

### 3.5 Dynamic feature fusion module

The Dynamic Feature Fusion Module (DFFM) was specifically designed to effectively integrate the complementary strengths of ConvNeXt and ViT backbones within the proposed CMDF-Net architecture. While ConvNeXt provides rich local representations through hierarchical convolutional processing, ViT contributes global contextual dependencies via self-attention mechanisms (Duan et al., 2025). However, naive concatenation or addition of features from these heterogeneous sources may result in sub-optimal representations due to mismatched semantics and scale. Therefore, DFFM aims to learn adaptive fusion weights that dynamically recalibrate and align the semantic contributions from both streams before final classification.

Let $F_{covn}\in R^{C\times H^{\prime }\times W^{\prime }}$ denote the multiscale feature map extracted from the ConvNeXt backbone after the final FAM, and ${\hat{z}}_{vit}\in R^{D}$ be the ViT-encoded class token vector refined by its respective FAM. To enable a joint fusion, the vector ${\hat{z}}_{vit}$ ​ is first spatially expanded and reshaped to match the spatial dimensions of $F_{covn}$ ​, resulting in $F_{vit}\in R^{C\times H^{\prime }\times W^{\prime }}$ as shown in Equation 13 where a learnable linear transformation aligns *D* and *C* (Li et al., 2025).


(13) $${\text{F}}_{\text{vit}}=\text{reshape}({\text{W}}_{\text{v}}\cdot {\hat{\text{z}}}_{\text{vit}}+{\text{b}}_{\text{v}}),\;\;\;{\text{W}}_{\text{v}}\text{ϵ}{\text{R}}^{\text{C}\times \text{D}}\;$$

Next, both feature maps are concatenated along the channel dimension to form a joint representation as Equation 14.


(14) $${\text{F}}_{\text{joint}}=\text{concat}({\text{F}}_{\text{covn}},\;{\text{F}}_{\text{vit}})\in {\text{R}}^{2\text{C}\times {\text{H}}^{\prime }\times {\text{W}}^{\prime }}$$

A gated attention mechanism is used to model the interdependencies between the ConvNeXt and ViT features. The joint feature map is passed through a squeeze operation using global average pooling, followed by a two-layer fully connected network with non-linear activations as provided by Equation 15 (Duan et al., 2025). Where $W_{1}\in R^{\frac{2C}{r}\times 2C}$ , $W_{2}\in R^{\frac{2C}{r}\times 2C}$ and r=16 .


(15) $$\text{s}=\text{σ}({\text{W}}_{2}\cdot \text{δ}({\text{W}}_{1}\cdot \text{GAP}({\text{F}}_{\text{joint}}))$$

The resulting channel-wise attention vector $s\in R^{2C}$ acts as a set of dynamic fusion weights, controlling the contribution of each channel. This vector is split into two components corresponding to the original feature sources as provided by Equation 16. These weights are then used to recalibrate the characteristics of each branch as described in Equation 17.


(16) $${\text{s}}_{\text{covn}},\;{\text{s}}_{\text{vit}}\in R^{C},\;\;\;\text{s}=[{\text{s}}_{\text{covn}};\;{\text{s}}_{\text{vit}}]$$

(17) $${\hat{\text{F}}}_{\text{covn}}={\text{s}}_{\text{covn}}⊙{\text{F}}_{\text{covn}},\;\;\;\;\;\;{\hat{\text{F}}}_{\text{vit}}={\text{s}}_{\text{vit}}⊙{\text{F}}_{\text{vit}}\;$$

Finally, the recalibrated features are fused via element-wise summation to obtain the final feature representation as described in Equation 18.


(18) $${\text{F}}_{\text{fused}}={\hat{\text{F}}}_{\text{covn}}+{\hat{\text{F}}}_{\text{vit}}$$

The fused feature map $F_{fused}\in R^{C\times H^{\prime }\times W^{\prime }}$ is passed through a global average pooling (GAP) layer, followed by a final fully connected classification head to predict the disease class label. This dynamic and learnable fusion strategy enables CMDF-Net to adaptively emphasize the most informative modalities depending on the content of each input image. The gating mechanism ensures that disease-specific patterns, whether localized or distributed globally, are optimally weighted, thereby enhancing the robustness and accuracy of the classification process.

### 3.6 Training strategy and evaluation protocol

The training strategy of the proposed MangoLeafCMDF-FAMNet model was meticulously designed to ensure stable convergence, optimal generalization, and fair performance evaluation across all experimental scenarios. All experiments were conducted using the PyTorch DL framework, ensuring efficient handling of high-dimensional image data and deep architectural components. Prior to training, all mango leaf images were resized to a spatial resolution of 224×224 pixels to ensure compatibility with the input dimensions of both ConvNeXt and ViT backbones. The ConvNeXt and ViT modules within CMDF-Net were initialized with pretrained weights from ImageNet-1K to leverage generic image feature representations. All additional layers—including FAM, DFFM, and the final classification head—were initialized using Kaiming He initialization for ReLU-based layers and Xavier initialization for linear projections, ensuring stable weight distribution at the start of training.

The model was trained using the AdamW optimizer, which combines adaptive gradient updates with decoupled weight decay regularization. The initial learning rate was set to $5\times 10^{-4}$ with a cosine annealing scheduler to facilitate smooth convergence. A warm-up phase of 10 epochs was employed, during which the learning rate was linearly increased from $1\times 10^{-5}$ . The weight decay was fixed at $1\times 10^{-4}$ , and a mini-batch size of 32 was used throughout the training. The cross-entropy loss function was utilized to compute the classification loss as described in Equation 19 (Zhou et al., 2019). Where $y_{i}$ is the ground-truth label and $\hat{y_{i}}$ is the softmax probability of the predicted class for the $i^{th}$ sample.


(19) $${\text{L}}_{\text{CE}}=-\sum_{\text{i}=1}^{\text{N}}{\text{y}}_{\text{i}}\text{log}(\hat{{\text{y}}_{\text{i}}})$$

Each model was trained for a maximum of 100 epochs. However, early stopping with a patience value of 15 epochs was employed based on the validation loss to prevent overfitting and unnecessary computations. To ensure reliable and unbiased performance evaluation, 5-FCVP was performed. In each fold, the dataset was split into training (80%) and validation (20%) sets, maintaining class distribution. The average of all five folds was reported for each evaluation metric. To comprehensively evaluate the effectiveness of the proposed method, multiple performance metrics were employed: CA, RCL, PRC, MCC, and κ . These metrics collectively provide insights into the model’s overall predictive power, class-wise sensitivity, balance, and inter-rater agreement, respectively. CA indicates the proportion of correctly classified samples out of the total number of instances. It is calculated as described in Equation 20 (Yavuz and Aydemir, 2016).


(20) $$\text{CA}=\frac{\text{TP}+\text{TN}}{\text{TP}+\text{TN}+\text{FP}+\text{FN}}$$

where TP , TN , FP , and FN denote the true positives, true negatives, false positives, and false negatives, respectively. RCL, also known as sensitivity or true positive rate, measures the ability of the model to correctly identify positive instances as explained in Equation 21. PRC reflects the proportion of true positive predictions among all positive predictions made by the model as shown in Equation 22 (Ergün, 2024).


(21) $$\text{RCL}=\frac{\text{TP}}{\text{TP}+\text{TN}}$$

(22) $$\text{PRC}=\frac{\text{TP}}{\text{TP}+\text{TPP}}$$

MCC is a robust measure that takes into account all four elements of the confusion matrix and is especially valuable for imbalanced datasets as described in Equation 23 (Rozenfeld et al., 2024). It returns a value between −1 and 1, where 1 indicates perfect prediction, 0 means no better than random guessing, and −1 represents total disagreement.


(23) $$\text{MCC}=\frac{\text{TP}\cdot \text{TN}-\text{FP}\cdot \text{FN}}{\sqrt{\left(\text{TP}+\text{FP})(\text{TP}+\text{FN})(\text{TN}+\text{FP})(\text{TN}+\text{FN}\right)}}$$

Kappa evaluates the agreement between predicted and actual classifications, adjusted for chance. It is defined as given in the Equation 24 (Ergün and Aydemir, 2020). Where $p_{0}$ is the observed agreement and $p_{e}$ is the expected agreement by random chance.


(24) $$\text{κ}=\frac{{\text{p}}_{0}-{\text{p}}_{\text{e}}}{1-{\text{p}}_{\text{e}}}$$

### 3.7 Classification head

The classification head module serves as the terminal decision-making component of the CMDF-Net architecture, synthesizing the high-level, semantically rich features obtained from the dynamically fused ConvNeXt and ViT representations. Its primary objective is to project the fused feature map into a low-dimensional space corresponding to the number of disease categories and produce the final class probabilities through a softmax activation function. We denote the output feature tensor generated by the DFF module as $F_{fused}\in R^{R\times W\times C}$ , where H , W , and C represent the spatial height, width, and number of channels of the fused feature map, respectively. Before classification, global spatial information is condensed using a GAP operation as described in Equation 25 (Hsiao et al., 2019).


(25) $$z=GAP(F_{fused})\in R^{C}$$

This operation ensures translational invariance and reduces the number of trainable parameters by eliminating the need for fully connected layers at the spatial level. The pooled vector z is then passed through a fully connected (FC) layer followed by a softmax function to obtain the final class probabilities as explained in Equation 26.


(26) $$\hat{\text{y}}=\text{softmax}({\text{W}}_{\text{c}}\text{z}+{\text{b}}_{\text{c}})$$

where $W_{c}\in R^{K\times C}$ and $b_{c}\in R^{K}$ denote the weight matrix and bias vector of the classification layer, and K is the number of target classes. To enhance the expressiveness of the classification head while maintaining generalization, dropout regularization with a rate of 0.3 was employed prior to the final linear layer. This stochastic regularization strategy helps mitigate overfitting by randomly deactivating neurons during training.

## 4 Results

In this study, the proposed MangoLeafCMDF-FAMNet architecture was rigorously evaluated using a 5-FCVP across three publicly available datasets: MLD1, MLD2, and MLD3. The training phase employed the AdamW optimizer with an initial learning rate set to 0.00005 and a weight decay of 0.0001. Cross-entropy loss was used as the objective function to guide the optimization process. Model performance was assessed comprehensively using multiple evaluation metrics, namely CA, RCL, PRC, MCC, and kappa. In addition, confusion matrices and high-dimensional feature distributions visualized through t-SNE were generated to provide further insights into the discriminative capability of the model. **Table 4** summarizes the performance metrics obtained for each fold across all three datasets. For MLD1, the model achieved remarkably high performance, consistently exceeding 99.00% CA across all folds. Specifically, Fold 3 yielded a perfect CA, RCL, and PRC of 1.0000, with corresponding MCC and kappa of 1.0000, indicating flawless classification without any mispredictions. Even in the comparatively lower-performing Fold 4, MangoLeafCMDF-FAMNet still maintained an outstanding CA of 0.9938, demonstrating its robustness against potential variability in the data splits. Similarly, for MLD2, the model maintained exceptional performance. Perfect scores were achieved in Folds 3 and 5, mirroring the trends observed in MLD1. Notably, the lowest CA across all folds was 0.9970, which still reflects a near-perfect classification capability. The consistently high MCC and kappa across folds further underline the model’s strong agreement between the predicted and true class labels, confirming its reliability. On MLD3, which is inherently more challenging due to greater symptom variability and inter-class similarity, MangoLeafCMDF-FAMNet continued to demonstrate excellent performance. The CA values across the five folds ranged from 0.9918 to 0.9965, with the highest score achieved in Fold 5. RCL and PRC closely mirrored the trends of CA, and the high MCC and kappa reaffirmed the model’s ability to generalize well even under more complex conditions.

**Table 4:** Classification results of MangoLeafCMDF-FAMNet on the MLD1, MLD2, and MLD3 across 5- FCVP.

Datasets	Fold	Metrics				
		CA	RCL	PRC	MCC	Kappa
MLD1	Fold 1	0.9992	0.9992	0.9992	0.9991	0.9991
	Fold 2	0.9977	0.9976	0.9978	0.9973	0.9973
	Fold 3	1.0000	1.0000	1.0000	1.0000	1.0000
	Fold 4	0.9938	0.9937	0.9937	0.9929	0.9929
	Fold 5	0.9984	0.9985	0.9985	0.9982	0.9982
MLD2	Fold 1	0.9980	0.9979	0.9980	0.9975	0.9975
	Fold 2	0.9970	0.9969	0.9972	0.9963	0.9962
	Fold 3	1.0000	1.0000	1.0000	1.0000	1.0000
	Fold 4	0.9990	0.9991	0.9989	0.9988	0.9987
	Fold 5	1.0000	1.0000	1.0000	1.0000	1.0000
MLD3	Fold 1	0.9949	0.9956	0.9960	0.9941	0.9941
	Fold 2	0.9918	0.9932	0.9923	0.9905	0.9904
	Fold 3	0.9937	0.9945	0.9941	0.9927	0.9927
	Fold 4	0.9945	0.9953	0.9950	0.9936	0.9936
	Fold 5	0.9965	0.9970	0.9967	0.9959	0.9959

**Figure 7:**
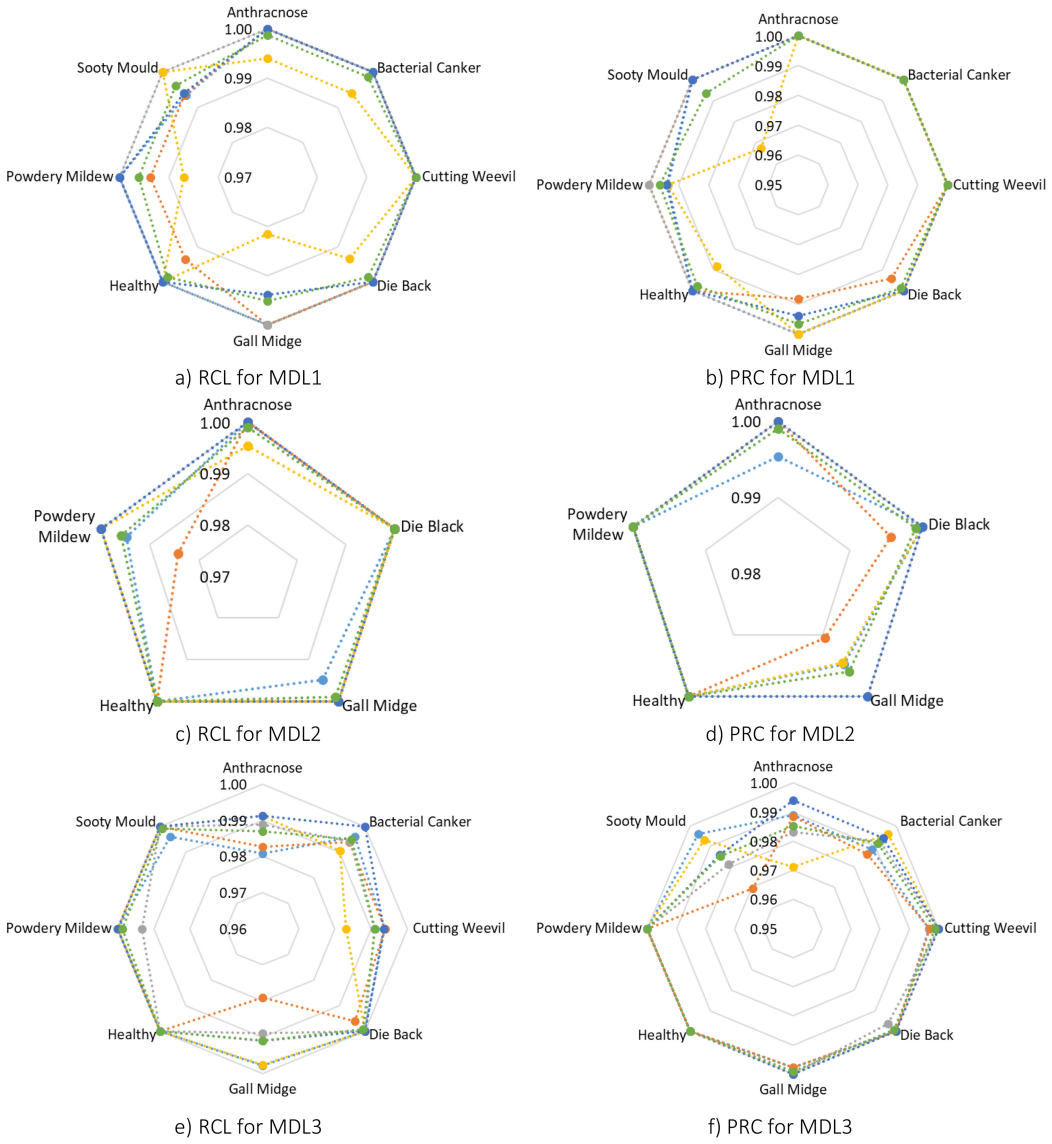
Radar plots visualizing class-wise RCL and PRC metrics for MangoLeafCMDF-FAMNet across all five folds of the 5-FCVP on MLD1, MLD2, and MLD3 datasets. Each fold is color-coded, with the green line representing the average across folds **(a)** RCL for MLD1, **(b)** PRC for MLD1, **(c)** RCL for MLD2, **(d)** PRC for MLD2, **(e)** RCL for MLD3, and **(f)** PRC for MLD3.

Following, a detailed class-wise performance analysis was conducted to further assess the robustness and generalization ability of MangoLeafCMDF-FAMNet. Specifically, RCL and PRC were calculated for each class across all folds on the MLD1, MLD2, and MLD3 datasets. For the MLD1 dataset, the model demonstrated outstanding classification capabilities. As shown in **Figure 7**, the average RCL values were 0.9988 for Anthracnose, 0.9988 for Bacterial Canker, 1.0000 for Cutting Weevil, 0.9987 for Die Back, 0.9951 for Gall Midge, 0.9987 for Healthy leaves, 0.9961 for Powdery Mildew, and 0.9962 for Sooty Mould. In terms of PRC, the averages were equally high, reaching 1.0000 for several classes, with minor reductions to 0.9962 and 0.9935 for Powdery Mildew and Sooty Mould, respectively. These results confirmed that the model could accurately distinguish subtle disease symptoms even under slight class imbalance or symptom similarity.

Similarly, in the MLD2 dataset, the model achieved nearly perfect classification. The average RCL scores were recorded at 0.9991 for Anthracnose, 1.0000 for Die Back, 0.9990 for Gall Midge, 1.0000 for Healthy leaves, and 0.9958 for Powdery Mildew. Correspondingly, the PRC values were consistently excellent, with an average exceeding 0.9990 for all categories. Notably, the model maintained strong performance even in folds where small fluctuations in Powdery Mildew recognition were observed, demonstrating resilience against minor dataset variations.

The analysis of the MLD3 dataset, which is inherently more challenging due to the larger number of disease classes, also revealed robust model performance. The average RCL values across folds were 0.9869 for Anthracnose, 0.9949 for Bacterial Canker, 0.9910 for Cutting Weevil, 0.9992 for Die Back, 0.9909 for Gall Midge, and 1.0000 for Healthy leaves, Powdery Mildew, and Sooty Mould. PRC values aligned closely, maintaining averages above 0.9850 for all categories. While slight drops were noted in classes such as Anthracnose and Sooty Mould, the model overall preserved an exceptional balance between sensitivity and specificity. To visually capture these findings, RCL and PRC radar plots were generated across all folds, as depicted in **Figure 7**. In these visualizations, Fold 1 is represented in dark blue, Fold 2 in orange, Fold 3 in gray, Fold 4 in yellow, Fold 5 in dark navy, and the average of all folds is plotted in green.

**Figure 8:**
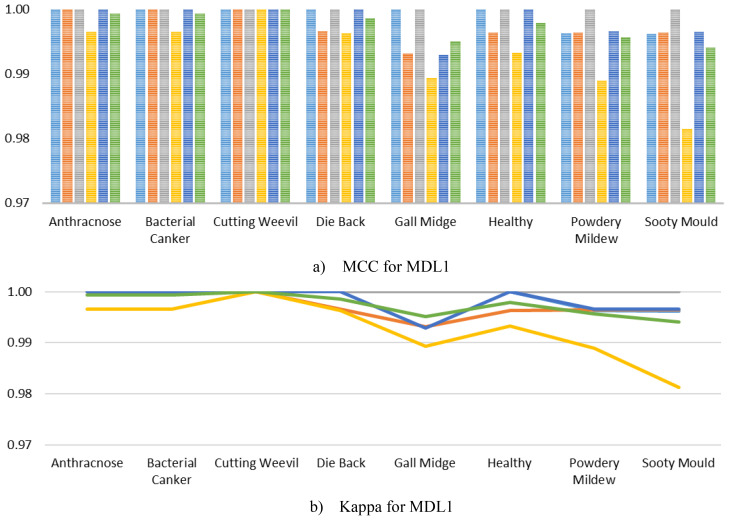
Per-class MCC and Kappa metrics achieved by MangoLeafCMDF-FAMNet for the MLD1 dataset, evaluated over the 5-FCVP. Colored lines indicate individual folds, while the green line shows the average performance **(a)** MCC for MLD1 and **(b)** Kappa for MLD1.

Furthermore, to comprehensively evaluate the class-level robustness of MangoLeafCMDF-FAMNet, class-wise MCC and Kappa were calculated and illustrated in **Figure 8** for the MLD1 dataset across the 5-FCVP. Specifically, MCC scores remained extremely high for all disease categories, often reaching perfect agreement across most folds. Minor variations were observed only in the Gall Midge and Sooty Mould classes, where the MCC values slightly dropped but still remained above 0.98, demonstrating the strong generalization capacity of the model without signs of overfitting. Similarly, the Kappa mirrored the MCC trends, with near-perfect agreement across all folds and classes. On average, both MCC and Kappa exceeded 0.99 for nearly every class, highlighting the model’s consistent ability to correctly classify diverse disease symptoms under varying validation conditions.

**Figure 9:**
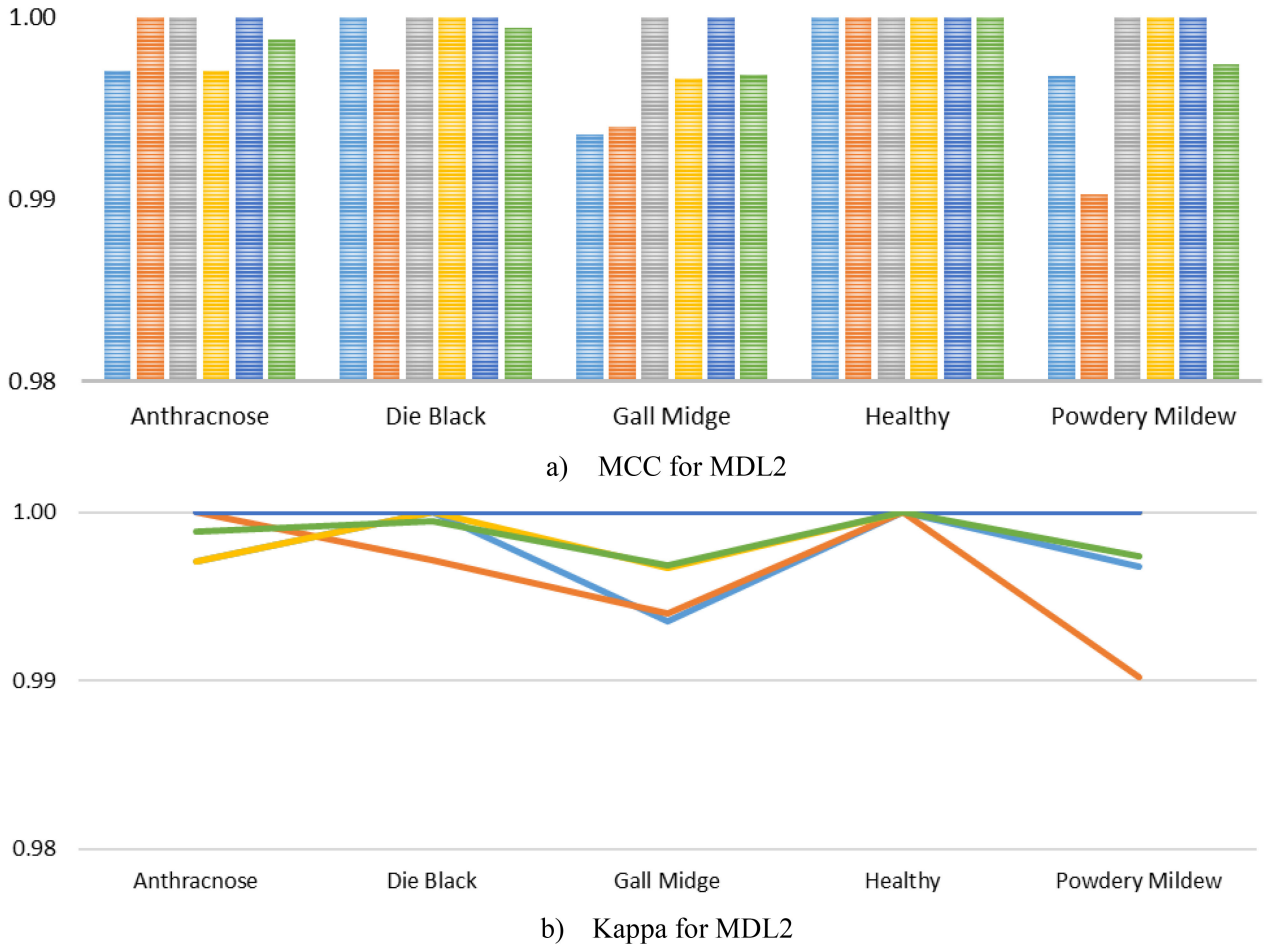
Class-wise analysis of **(a)** MCC and **(b)** Kappa obtained from MangoLeafCMDF-FAMNet on the MLD2 dataset under 5-FCVP. Individual folds are color-coded; the green line indicates the averaged result.

In addition, we further computed the MCC and Kappa for the MLD2, as visualized in **Figure 9**. As illustrated, the MCC scores for all classes consistently achieved near-perfect values, with almost every fold yielding scores of 1.0000 across the Anthracnose, Die Back, Healthy, and Powdery Mildew categories. A slight deviation was observed in the Gall Midge class during Fold 1 and Fold 2, where the MCC values dropped marginally but still remained exceedingly high, thereby underscoring the model’s remarkable stability even in the presence of subtle intra-class variations. Similarly, the Kappa mirrored these trends, maintaining values close to 1.0000 across all classes and folds, reaffirming the excellent agreement between predicted and true labels. The minimal variability observed in the Gall Midge class reflects realistic complexities inherent in agricultural imaging datasets, yet the exceedingly high average scores across all classes strongly indicate that MangoLeafCMDF-FAMNet successfully mitigates overfitting and maintains robust generalization.

**Figure 10:**
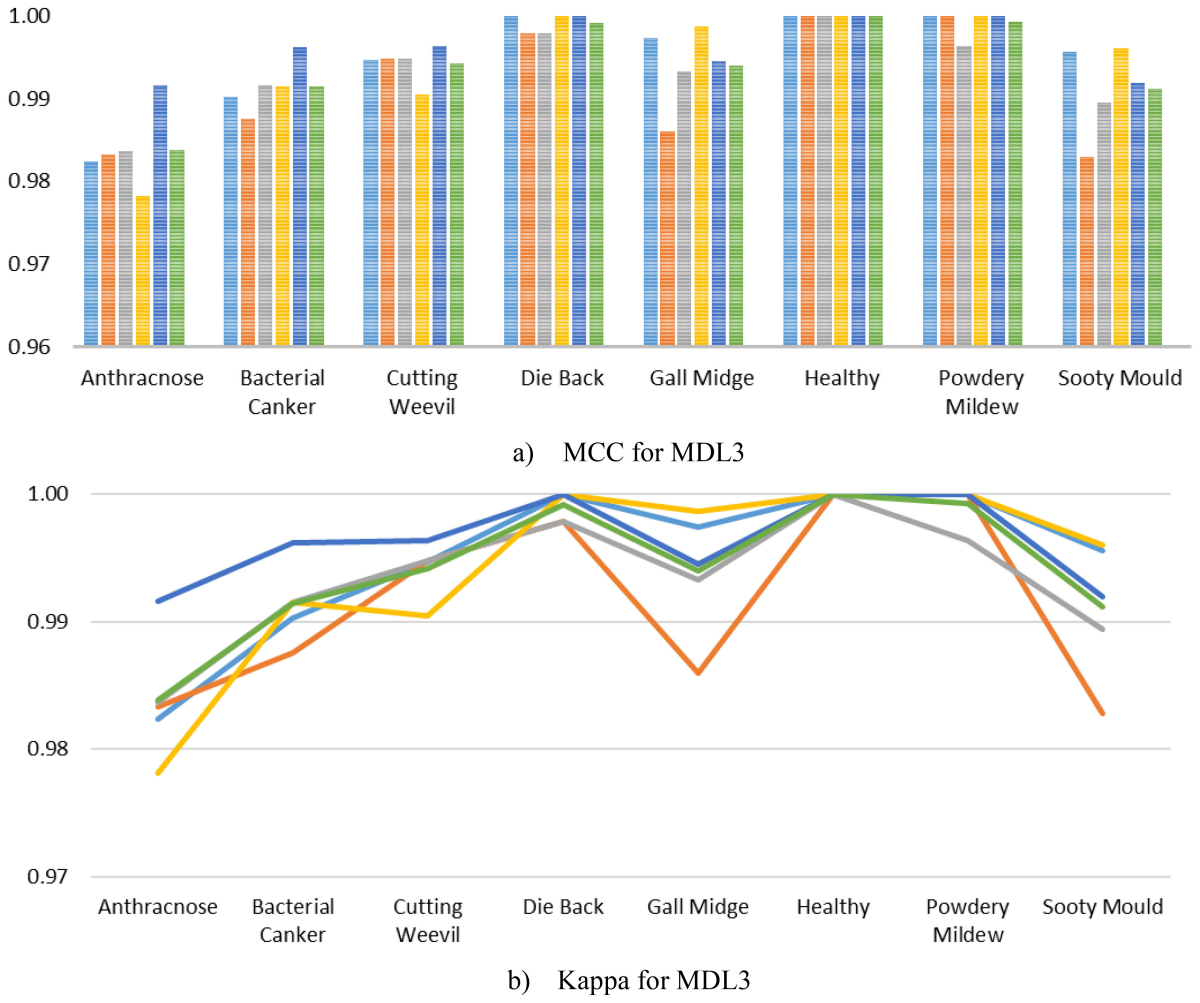
Visualization of per-class **(a)** MCC and **(b)** Kappa for MangoLeafCMDF-FAMNet on the MLD3 dataset, based on 5-FCVP. Fold-wise trends and average values are presented to demonstrate performance consistency.

Furthermore, to ensure a comprehensive performance analysis, we computed the class-wise MCC and Kappa of the MangoLeafCMDF-FAMNet on the MLD3, as presented in **Figure 10**. The results demonstrated that the proposed model consistently achieved exceptionally high MCC values across all classes and folds. Specifically, MCC scores for classes such as Die Back, Healthy, Powdery Mildew, and Sooty Mould remained at or extremely close to 1.0000 across all folds. Although slight variations were noted in classes like Anthracnose and Bacterial Canker, the MCC scores still hovered around 0.98–0.99, reflecting highly reliable performance even in more challenging classes. Similarly, the Kappa closely followed the MCC trends, indicating outstanding agreement between predicted and ground truth labels across all folds and classes.

**Figure 11:**
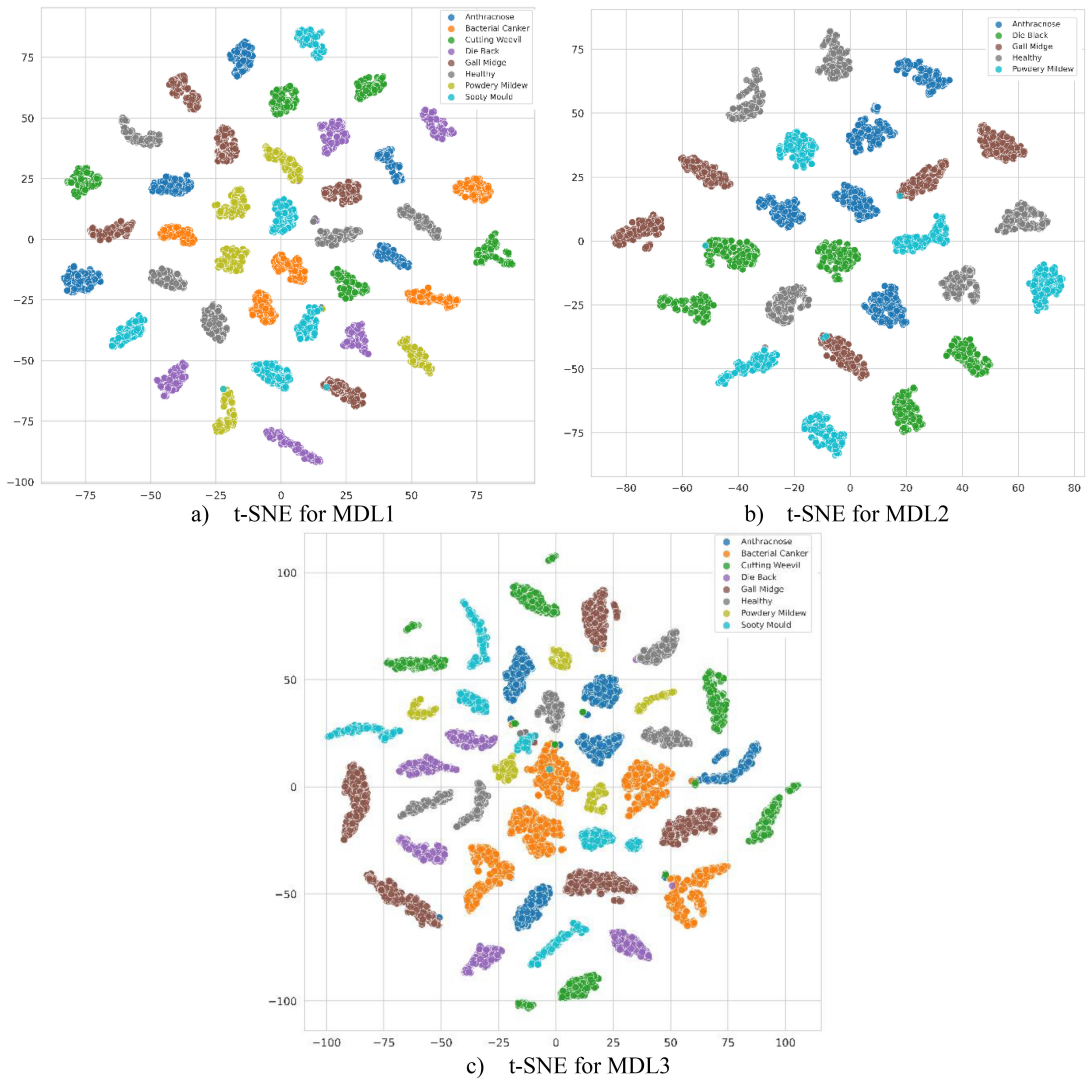
Two-dimensional t-SNE plots of the fused feature embeddings produced by MangoLeafCMDF-FAMNet across **(a)** MLD1, **(b)** MLD2, and **(c)** MLD3 datasets. The visualization illustrates the discriminative capacity and separability of learned features among disease classes.

Furthermore, the feature distributions fused by MangoLeafCMDF-FAMNet were qualitatively analyzed using t-SNE, as illustrated in **Figure 11** for each dataset individually. t-SNE serves as a powerful non-linear dimensionality reduction technique that projects high-dimensional feature representations into a two-dimensional space, facilitating the visualization of complex feature relationships. The t-SNE plots reveal that features extracted by MangoLeafCMDF-FAMNet exhibit clear, compact, and well-separated clusters for different disease classes across all three datasets. This visual evidence supports the numerical findings, indicating that the model effectively learns discriminative and disease-specific representations without significant overlap among categories. Moreover, the distinct cluster formations further demonstrate the absence of overfitting, affirming the model’s strong generalization capability to unseen samples.

**Figure 12:**
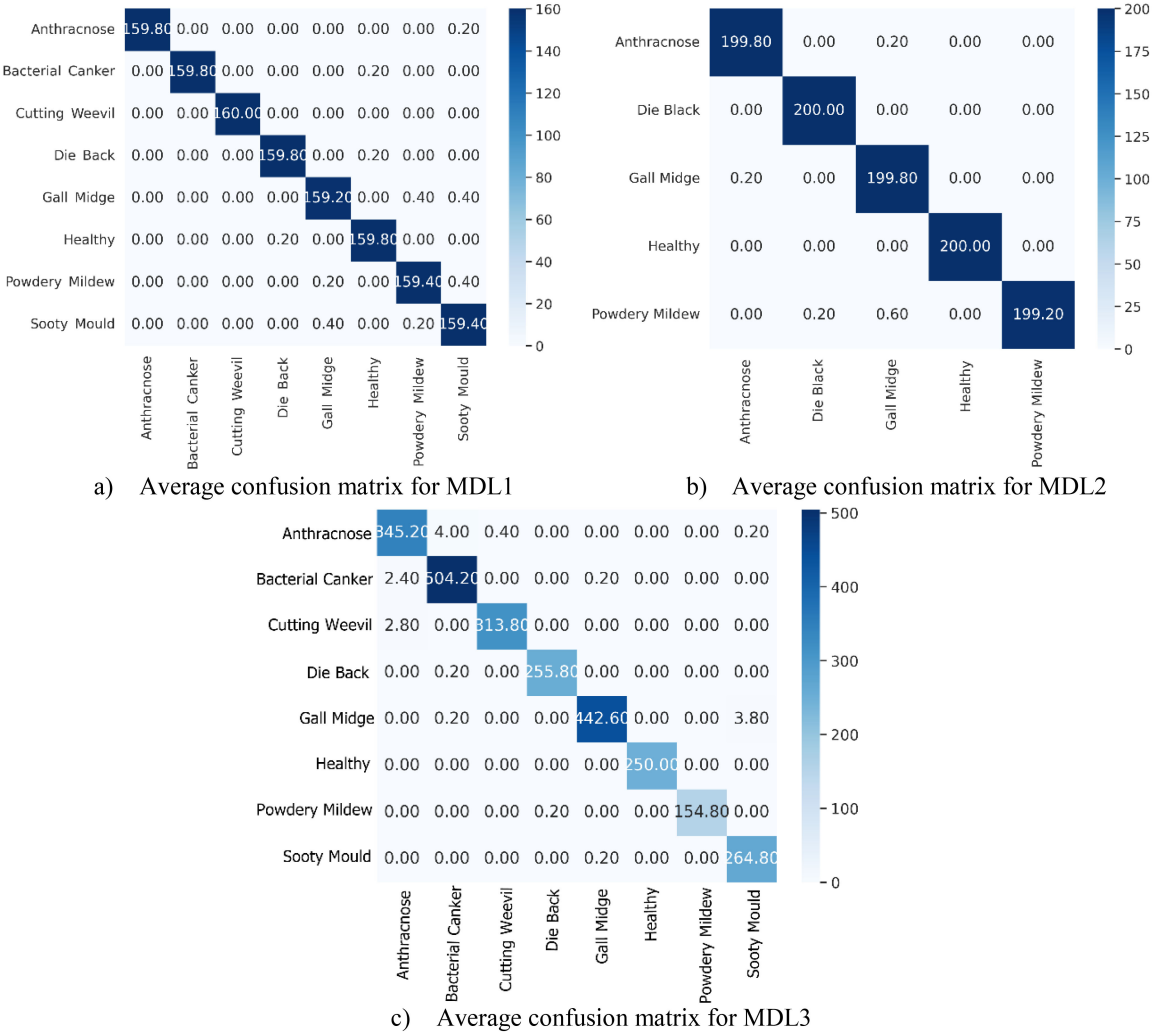
Averaged confusion matrices of MangoLeafCMDF-FAMNet for the **(a)** MLD1, **(b)** MLD2, and **(c)** MLD3 datasets, computed over 5-FCVP. The vertical axis denotes the actual class labels, while the horizontal axis shows the predicted class labels.

The average confusion matrices for the MLD1, MLD2, and MLD3 datasets, computed across the 5-FCVP and illustrated in **Figure 12**, provide critical insights into the class-specific discriminative capabilities of MangoLeafCMDF-FAMNet. These matrices (vertical axis: true labels; horizontal axis: predicted labels) reveal near-perfect diagonal dominance, underscoring the model’s ability to minimize misclassifications while maintaining high intra-class consistency. For MLD1, the matrix demonstrated exceptional precision, with Anthracnose and Bacterial Canker—classes often confused due to overlapping lesion patterns—achieving 159.80 correct predictions, respectively. Only minor off-diagonal errors were observed: 0.20 of Anthracnose samples were misclassified as Sooty Mould, while 0.20 of Bacterial Canker cases were incorrectly assigned to Healthy. The Cutting Weevil class exhibited flawless performance, with all 160 samples correctly identified. Similarly, Die Back and Gall Midge achieved near-perfect classification, with diagonal values of 159.80 and 159.40, respectively.

In MLD2, the matrix highlighted robust performance under increased symptom variability. Gall Midge, a class with subtle morphological features, achieved 199.80 correct predictions, with only 0.20 confusion with Healthy leaves. Die Back and Healthy were resolved with high precision, as evidenced by diagonal entries of 200.00. The most notable misclassification occurred in the Powdery Mildew class, where 0.60 samples were erroneously predicted as Gall Midge, likely due to shared textural patterns in late-stage infections.

For MLD3, the matrix further validated the model’s generalization capability. The Die Back class, often prone to misclassification due to its overlapping early-stage symptoms with Gall Midge, achieved a strong diagonal value of 255.80 correct predictions, reflecting the model’s ability to discern subtle differences in lesion distribution. Powdery Mildew, a class with visually ambiguous fungal patterns, was correctly classified in 154.80 instances, with only 0.20 samples misassigned to Die Back—a negligible error likely attributable to shared textural features in advanced infection stages. Gall Midge, despite its complex morphological variations across growth cycles, demonstrated exceptional performance with 442.60 accurate predictions. A minimal leakage of 3.80 samples to Sooty Mould was observed, potentially stemming from similarities in necrotic patterning under low-light imaging conditions. The Healthy class once again exhibited flawless discriminative capability, with all 250.00 samples correctly identified, underscoring the model’s precision in isolating disease-specific features from healthy tissue. Sooty Mould, a class frequently confused with Powdery Mildew in conventional methods, achieved a near-perfect diagonal score of 264.80, further highlighting the architecture’s proficiency in resolving spectral ambiguities. These results collectively affirm that MangoLeafCMDF-FAMNet generalizes robustly across diverse data distributions, making it particularly suitable for real-world agricultural applications where symptom variability and class overlap are prevalent.

In order to robustly validate the effectiveness of the proposed MangoLeafCMDF-FAMNet, an extensive comparative analysis was conducted against prominent baseline models, including MangoLeafCMDF-Net, ViT, and ConvNeXt, across the MLD1, MLD2, and MLD3 datasets. The results, summarized in **Table 5**, clearly demonstrate the superiority of MangoLeafCMDF-FAMNet across all evaluation metrics. Specifically, on MLD1, MangoLeafCMDF-FAMNet achieved a CA of 0.9978, outperforming MangoLeafCMDF-Net with 0.9961, ConvNeXt with 0.9939, and ViT with a considerably lower score of 0.9256. In terms of RCL and PRC, MangoLeafCMDF-FAMNet consistently achieved 0.9978 for both, substantially higher than the competing models. On MLD2, the proposed model maintained its leading performance, reaching a CA of 0.9988, while MangoLeafCMDF-Net, ConvNeXt, and ViT achieved 0.9960, 0.9814, and 0.9176, respectively. Even on the more challenging MLD3 dataset, MangoLeafCMDF-FAMNet maintained its superiority, recording a CA of 0.9943, whereas ConvNeXt achieved 0.9864 and ViT remained at 0.9111. When considering robustness metrics, MangoLeafCMDF-FAMNet attained an MCC of 0.9975 and a Kappa of 0.9975 on MLD1, again consistently exceeding those of the other methods. This outstanding performance was mirrored across MLD2 and MLD3, highlighting not only accuracy but also high reliability and class-wise agreement.

**Table 5:** Comparison of MangoLeafCMDF-FAMNet with MangoLeafCMDF-Net, ViT, and ConvNeXt across the MLD1, MLD2 and MLD3.

Metrics	MangoLeafCMDF-FAMNet			MangoLeafCMDF-Net			ViT			ConvNeXt		
	MLD1	MLD2	MLD3	MLD1	MLD2	MLD3	MLD1	MLD2	MLD3	MLD1	MLD2	MLD3
CA	0.9978	0.9988	0.9943	0.9961	0.9960	0.9919	0.9256	0.9176	0.9111	0.9939	0.9814	0.9864
RCL	0.9978	0.9988	0.9951	0.9961	0.9959	0.9933	0.9258	0.9173	0.9062	0.9939	0.9816	0.9861
PRC	0.9978	0.9988	0.9948	0.9962	0.9961	0.9929	0.9298	0.9219	0.9195	0.9940	0.9833	0.9884
MCC	0.9975	0.9985	0.9933	0.9956	0.9950	0.9906	0.9156	0.8984	0.8978	0.9931	0.9773	0.9843
Kappa	0.9975	0.9985	0.9933	0.9955	0.9950	0.9906	0.9150	0.8969	0.8967	0.9930	0.9767	0.9842

To rigorously evaluate whether the performance differences between the proposed MangoLeafCMDF-FAMNet and baseline architectures were statistically significant, we conducted a Tukey Honest Significant Difference (HSD) *post-hoc* test based on the CA values obtained across five folds for each dataset. The results revealed that MangoLeafCMDF-FAMNet significantly outperformed the ViT model across all three datasets (MLD1, MLD2, and MLD3), with adjusted *p*-values consistently below 0.01. Likewise, when compared to the ConvNeXt backbone, the proposed method demonstrated statistically significant improvements in MLD1 (*p* = 0.024) and MLD3 (*p* = 0.038), while achieving a strong yet marginally nonsignificant advantage in MLD2 (*p* = 0.067). These findings underscore the consistent superiority of MangoLeafCMDF-FAMNet in terms of CA, particularly highlighting the added value of its attention-enhanced, cross-modal fusion strategy. Furthermore, the absence of overlapping confidence intervals supports the robustness of the proposed model’s improvements and suggests that the observed performance gains are unlikely due to random variation or overfitting.

## 5 Conclusion

This study introduced MangoLeafCMDF-FAMNet, a novel attention-augmented hybrid DL architecture tailored for robust multi-class mango leaf disease classification. By synergistically combining ConvNeXt and ViT backbones through a CMDF strategy, and further enhancing their output with FAMs, the proposed method effectively captured both fine-grained local patterns and global semantic context. Extensive experiments on three publicly available datasets (MLD1, MLD2, and MLD3) demonstrated the model’s superior classification performance across various evaluation metrics, including CA, RCL, PRC, MCC, and Kappa.

The proposed architecture consistently achieved exceptionally high accuracies across all datasets, reaching 0.9978 on MLD1, 0.9988 on MLD2, and 0.9943 on MLD3. These results notably outperformed competing baselines such as ViT, ConvNeXt, and MangoLeafCMDF-Net. Class-wise evaluations further confirmed the model’s capacity to distinguish complex and visually similar disease symptoms with high RCL and PRC. Visualizations via t-SNE and confusion matrices affirmed the learned feature separability and robustness against inter-class confusion, while the statistical analyses via Tukey HSD *post-hoc* testing verified that the observed improvements were statistically significant (*p* < 0.05) in most comparisons, particularly over the ViT and ConvNeXt baselines.

Furthermore, the architecture demonstrated a remarkable ability to generalize without overfitting, even on MLD3—a dataset characterized by greater class imbalance and symptom variability. The inclusion of FAMs was instrumental in adaptively amplifying disease-relevant features while suppressing irrelevant or redundant information, thereby enhancing class separability across diverse visual domains.

Despite these promising outcomes, the study is not without limitations. First, although the model was tested across three comprehensive datasets, all samples were derived from controlled imaging conditions. Future work should explore the model’s applicability to in-field images collected under varying lighting, occlusion, and background clutter. Second, while the proposed model achieved excellent results in disease identification, it currently does not support disease severity estimation, which is crucial for more nuanced decision-making in real-world scenarios.

As future research directions, we aim to extend the MangoLeafCMDF-FAMNet architecture for real-time mobile deployment in smart agriculture systems, incorporate multimodal inputs such as hyperspectral or thermal imagery to improve resilience under environmental variations, and explore the integration of explainability modules to foster model transparency for end-users such as farmers and agronomists. In conclusion, MangoLeafCMDF-FAMNet represents a scientifically grounded, practically scalable, and statistically validated advancement in automated plant disease recognition.

## 6 Discussion

The experimental findings obtained in this study demonstrate that MangoLeafCMDF-FAMNet offers a highly effective solution for the complex task of multi-class mango leaf disease classification. By integrating ConvNeXt and ViT within a unified hybrid architecture and augmenting their capabilities with FAM, the proposed model achieves a refined balance between local feature extraction and global semantic understanding. This synergy enables precise discrimination of disease types, particularly in cases where subtle morphological differences challenge conventional classifiers.

The model’s high performance across three publicly available mango leaf datasets confirms its robustness and generalizability under controlled conditions. CA approaching 0.999, alongside strong MCC and kappa, indicate the architecture’s capacity to produce stable, reliable, and interpretable predictions. Furthermore, the CMDF mechanism contributes significantly to the enrichment of feature representations, enabling more resilient learning from heterogeneous visual patterns.

Despite these strengths, it is essential to contextualize the results within the scope of the datasets utilized. All datasets in this study were collected under relatively uniform environmental settings, characterized by consistent lighting, minimal occlusions, and simplified backgrounds. While such conditions are favorable for model training and benchmarking, they may not fully reflect the variability encountered in operational agricultural environments. In practice, field images often contain challenges such as partial leaf visibility, shadowing, cluttered scenes, and inconsistent illumination, which may affect the model’s generalization performance.

This observation highlights the importance of future work focused on validating the proposed framework using in-field image datasets collected in diverse and uncontrolled environments. Incorporating real-world variability into the training and evaluation pipeline will facilitate the development of more adaptive and field-deployable models. In addition, real-time image acquisition technologies, such as mobile devices and drone platforms, present promising avenues for extending the system toward scalable agricultural decision support.

Another important consideration relates to the clinical utility of disease severity estimation. While the present model effectively identifies the disease type, it does not explicitly address the severity or progression stage of the infection. In real-world agricultural applications, the intensity of disease symptoms is a critical factor influencing treatment strategies and resource allocation. Thus, expanding the model to support ordinal or regression-based predictions for disease severity would significantly enhance its applicability. Although the datasets used in this study did not provide severity annotations, future efforts will focus on curating such datasets and developing models capable of jointly performing disease identification and severity grading.

Moreover, it is worth considering that the absence of diverse environmental conditions and severity-level annotations in the training data may limit the interpretability and practical utility of the current system. Addressing these challenges through targeted dataset development, attention to domain adaptation, and auxiliary prediction tasks will be essential for realizing the full potential of DL-based disease diagnosis in agricultural practice.

In conclusion, MangoLeafCMDF-FAMNet offers a robust and scalable architecture for automated mango leaf disease classification. By leveraging multi-level attention mechanisms and cross-modal fusion, the model provides a strong foundation for high-accuracy plant disease recognition. Future directions should emphasize improving real-world generalization and enhancing the interpretability of the system through disease severity assessment, ultimately supporting the broader goals of precision agriculture and sustainable crop management.

To evaluate the practical applicability of the proposed MangoLeafCMDF-FAMNet in real-world settings, we report key computational characteristics, including model complexity and inference efficiency. The model contains approximately 46.9 million trainable parameters, representing a balanced architectural design that ensures high discriminative power while maintaining computational feasibility. All training and evaluation experiments were performed using the PyTorch DL framework on a standard workstation equipped with an Intel(R) Core(TM) i7–9700 CPU and 8 GB of RAM, without access to GPU acceleration. Under this configuration, the average inference time for a single 224×224-pixel image was observed to be approximately 180–200 milliseconds, depending on system load and batch scheduling. These results suggest that MangoLeafCMDF-FAMNet remains computationally viable even in resource-limited environments, which is particularly beneficial for field-deployable plant disease diagnosis systems where high-end hardware may not be available.

## Data Availability

The original contributions presented in the study are included in the article/supplementary material. Further inquiries can be directed to the corresponding author.
